# Epidemiology of Chikungunya in the Americas

**DOI:** 10.1093/infdis/jiw390

**Published:** 2016-12-05

**Authors:** Sergio Yactayo, J. Erin Staples, Véronique Millot, Laurence Cibrelus, Pilar Ramon-Pardo

**Affiliations:** 1 Department of Pandemic and Epidemic Diseases, World Health Organization, Geneva, Switzerland; 2 Division of Vector-Borne Diseases, National Center for Emerging and Zoonotic Infectious Diseases, Centers for Disease Control and Prevention, Fort Collins, Colorado; 3 Department of International Health Regulations, Pan American Health Organization/World Health Organization, Washington D.C.

**Keywords:** chikungunya, epidemiology, arbovirus, emerging disease, Americas

## Abstract

Chikungunya virus (CHIKV) emerged in the Americas in late 2013 to cause substantial acute and chronic morbidity. About 1.1 million cases of chikungunya were reported within a year, including severe cases and deaths. The burden of chikungunya is unclear owing to inadequate disease surveillance and underdiagnosis. Virus evolution, globalization, and climate change may further CHIKV spread. No approved vaccine or antiviral therapeutics exist. Early detection and appropriate management could reduce the burden of severe atypical and chronic arthritic disease. Improved surveillance and risk assessment are needed to mitigate the impact of chikungunya.

Chikungunya virus (CHIKV) is an arthropod-borne alphavirus primarily transmitted by *Aedes* mosquitoes, which are endemic in tropical areas of Africa and Asia. It is also capable of causing explosive outbreaks of human disease in areas with no prior immunity, such as those in Europe and the Americas. Studies of prior outbreaks have determined that 10%–70% of persons in an affected area become infected [1, 2]. This potentially high infection rate is coupled with a high symptomatic attack rate among infected people, with 50%–97% developing clinical disease with fever and polyarthralgia [2–4], and yields large outbreaks of disease that often tax existing healthcare systems and public health infrastructure.

Serosurveys conducted during postepidemic phases detected immunoglobulin M/immunoglobulin G in 38.2% of surveyed populations in the Indian Ocean island of La Reunion [1], 75% in the Kenyan island of Lamu, 63% in Grande Comoro Island [2], and 90.4% in Suriname [5]. These findings suggest that the CHIKV was broadly transmitted during the outbreak. A total of 3%–22% of infected patients are asymptomatic or pauci-symptomatic [2, 4].

The recent expansion of CHIKV in the Americas highlights several of the features of this reemerging virus. It spreads rapidly, affecting populated areas with no prior immunity. Areas with outbreaks of other arboviruses, such as dengue virus and yellow fever virus, are at substantial risk of large CHIKV outbreaks since these viruses share similar ecology and vector transmission cycles with CHIKV. Although chikungunya is often described as a self-limited illness, rare but serious disease and death associated with CHIKV have been observed. In La Reunion, 51% of infected children had a global neurodevelopmental delay, compared with 15*%* of uninfected children [6]. Nevertheless the neurotropism of chikungunya has not been completely defined, and animal studies show some inconsistencies in the capacity for CHIKV to invade the brain parenchyma [7]. In addition, outbreaks from other regions, such as La Reunion, highlighted that CHIKV is capable of undergoing mutation that increases its infectivity for *Aedes albopictus*, which allows the virus to spread into areas where there is minimal population immunity. Nevertheless, there is no evidence that human clinical presentation in the Americas differs from that in La Reunion [8].

## CHIKUNGUNYA IN THE PAST

Before December 2013, local CHIKV transmission had not been identified in the Americas. The potential first reports of a chikungunya-like illness were recorded in 1823, in Zanzibar, where it was called *kidinga pepo*, a Swahili term meaning a disease characterized by a sudden cramp-like seizure. However, it was only years later that an epidemic of this disease was described in Zanzibar by Christie, in an 1872 issue of the *British Medical Journal* [9]. In 1827 and 1828, a chikungunya-like disease was described in St. Thomas Island in the Caribbean and later affected New Orleans, Louisiana, and part of South Carolina in the United States [9].

In 1928, Dumaresq, who observed a “denga” epidemic in New Orleans, explained how the chikungunya-like disease was imported into Havana, Cuba, by slaves coming from Africa. As recently recalled by Halstead, Dumaresq provided a precise clinical description of the disease as “[a] person on the disappearance of this fever would attempt to rise from bed, feeling not much loss of strength, and a consciousness of being able to move about and attend to a little to business; but how egregiously would he be mistaken when he assumed the upright posture! The joints felt as if fettered or anchylosed, and the advance of one foot or leg beyond the other, would cost more pain and effort than the purpose for which it may have been advanced was worth, —aye,—a thousand times told!” [9, 10].

The word chikungunya appeared for the first time in Tanzania in the 20th century, where in Makonde language it means “to walk bent over” and refers to the stooped posture of patients experiencing the joint pains that characterize this dengue-like infection [9]. Following the first identification of CHIKV in Tanzania, in 1952, the virus was then identified to cause sporadic cases of disease and localized outbreaks in Africa and parts of Asia. In 2004, the virus seemed to reemerge, initially in Kenya, before spreading through countries in and around the Indian Ocean, resulting in millions of disease cases and the first description of severe disease and death related to this virus. Age-related clinical presentation was reported in Suriname [5].

These large-scale outbreaks eventually led to the introduction of CHIKV into more-temperate locations, with Italy reporting a local outbreak of CHIKV infection in 2007. Aggressive vector control activities combined with cooler temperatures eventually resulted in cessation of CHIKV transmission in Italy. The large-scale outbreaks also led to an increase of travel-related cases in the Americas. From 1995 to 2005, only 3 cases of chikungunya were identified in US travelers. From 2006 through 2013, 28 cases of CHIKV infection per year were reported among travelers in the United States, with most of the infections acquired in areas of Asia where there were large-scale outbreaks. Between 2013 and 2015, at least 3467 imported cases were reported in the United States [2].

## CURRENT CHIKUNGUNYA SITUATION IN THE AMERICAS

The emergence (or, most likely, reemergence) of CHIKV in the Americas was announced in December 2013, when the French National Reference Center for Arboviruses diagnosed the first local chikungunya cases in Saint Martin [9–11]. At that time, this area was facing a concomitant dengue virus outbreak and the first chikungunya cases were initially identified as dengue-like fever. Chikungunya and dengue have a similar clinical presentation, which makes the clinical diagnosis difficult. Patients with either disease can present with fever, myalgia, headache, arthralgia, and rash. The different symptoms' frequency and description of the clinical presentation may help, especially when several cases occur in identical locations and where scarce laboratory capacities limit diagnostic options [2].

**Figure 1.**
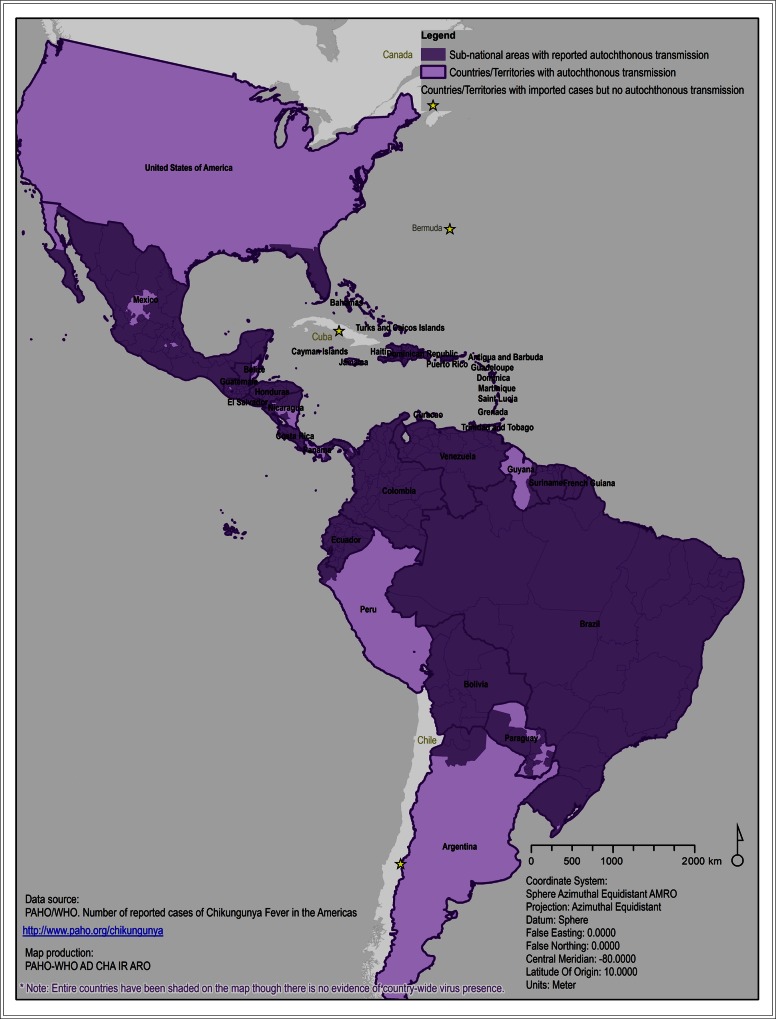
Areas with reported autochtonous transmission from 2013 to 2015.

Since it was first reported in Saint Martin, chikungunya has spread to 45 countries and territories in North, Central, and South America (Figure 1), causing >2.9 million suspected and confirmed cases and 296 deaths as of late July 2016 [12]. As in many other vector-borne diseases, outbreaks were mainly reported during the rainy season, when the density of mosquitoes is maximal. High disease attack rates were reported in areas such as in the Dominican Republic, (41%) or Suriname (90.4%), and the transmission peak was reached within 3 months [13, 14]. Although comparable to the attack rates noted in Malaysia (55.6%) and India (37.5%), these are higher than the attack rate observed in La Reunion (16.5%) [1, 2, 4]. Differences in attack rates are expected and may be explained by factors such as surveillance practices; season of CHIKV introduction into a country or a region, which has a direct effect on vector density and activity; vector control measures; and lifestyle differences (eg, use of air conditioning; Table 1). These factors may result in underestimation or overestimation of attack rates during an epidemic.

**Table 1.** Epidemiological Indicators for Chikungunya Outbreaks in La Reunion and Asia and in the Americas

Indicator	La Reunion and Asia	Americas
Attack rate, %	16.5–55.6 [1, 2, 4]	41 [13]
Cases that are asymptomatic, %	3–22 [2, 4]	Not available
Seroprevalence rate, %	38.2–75 [1, 15]	90.4 [5]
Case-fatality ratio, %	<1 [16]	<1 [17]
At-risk group(s)	Newborns, >55 y, underlying clinical conditions [18]	>45 y, underlying clinical conditions [19]
Persisting CHIK disease	In up to 64% for >1 y after initial infection; in 12% 3–5 y later [20, 21]	In 48% for a median time of 20 mo [22]; in 22% for 6 mo [5]
Mother-to-child vertical transmission rate, %	48.7 [23]	Not available

The illness caused by CHIKV is typically not fatal, with <1% of individuals with chikungunya in Colombia and Venezuela dying [17]. However, the real proportion of CHIKV-associated deaths is unknown and remains under investigation. The disease has also been associated with significant acute and long-term morbidity. According to a recent model based on the number of patients with acute chikungunya reported in 2014 in the Americas (n = 855 890 [12]), an estimated 385 835–429 058 persons (48%; 95% confidence interval, 45%–50%) will develop postchikungunya chronic inflammatory rheumatism in a median time of 20 months [22]. Also, the economic cost of the disease, including lost wages, in the Americas is expected to be overwhelming. A study in Venezuela has estimated the cost of chikungunya in that country to be >1 billion dollars [24].

It is currently unclear to what degree CHIKV will persist and continue to circulate in the Americas. Most of the disease that has been reported to date was likely a result of human-to-mosquito transmission. An enzootic cycle can be established among the nonhuman primates (NHPs) in Africa and the Americas, depending on a species’ susceptibility to the development of chikungunya viremia. However, no important clinical manifestations of the disease have been observed in NHPs so far, compared with yellow fever [25, 26]. Studies are necessary to assess the role of NHPs but also of other potential reservoirs (such as rodents, birds, or other vertebrates yet to be identified) in maintaining CHIKV in the environment.

## CLINICAL CASE DEFINITION

Newer chikungunya case definitions were recently proposed for the Americas. The definitions include 4 categories of cases: (1) acute clinical cases, characterized by fever (temperature, >38.5°C [101.3°F]) and joint pain (usually incapacitating) with acute onset and/or epidemiological and laboratory criteria; (2) atypical cases, characterized by clinical cases of laboratory-confirmed CHIKV accompanied by other manifestations (ie, neurological, cardiovascular, dermatological, ophthalmological, hepatic, renal, respiratory, or hematological conditions); (3) severe acute cases, characterized by clinical cases of laboratory-confirmed CHIKV presenting with dysfunction of at least 1 organ or system that threatens life and requires hospitalization; and (4) suspected/confirmed chronic cases, characterized by previous clinical diagnosis of chikungunya 12 weeks after symptom onset and presentation with at least 1 rheumatologic (joint) manifestation (ie, pain, rigidity, or edema) that is continuous or recurrent [14].

## ECOLOGICAL AND ENVIRONMENTAL FACTORS

Disease spread cannot be entirely determined by risk factors for chikungunya, but some of them may have strong influences on human (population density, activity, and migration) and vector (type, density, and infectivity) populations.

Ecological and environmental factors provide complementary information for risk assessment. Access to data and information on the environmental and demographic conditions in the area of interest, both historically and at the time of the study, may support the interpretation and analyses of CHIKV activity. The interpretation of human serosurvey findings and entomological assessments is helped by using ecological and environmental indicators to explain how and why these indicators influence the changes in vector and human populations.

Some ecological and environment indicators can potentially impact CHIKV activity [27], such as (i) temperature, which has an impact on human migration and the range of the mosquito habitat; (ii) elevation, which limits the reach of mosquito habitat to altitudes of <2300 m; (iii) rainfall and vegetation, which can change the range of mosquito habitat; (iv) land use and industry, which can influence human demographic changes and their accompanying effects on CHIKV; and (v) population movements due to migration, tourism, and trade within and across borders.

## THE FUTURE OF CHIKUNGUNYA IN THE AMERICAS

As of late 2015, CHIKV continues to circulate and cause disease in many of the countries in the Americas. As noted above, it is unclear whether CHIKV will establish an enzootic cycle in the Americas that would facilitate continual occurrence of CHIKV outbreaks as immunologically naive humans encroach on this cycle and become infected. If CHIKV develops an enzootic cycle like that of yellow fever virus, it may be possible for early detection to rely not only on traditional human surveillance, but also on ecologic surveillance, such as epizootic and entomological surveillance for vector density and infectivity [26]. Early risk detection using selected ecological and environmental indicators such as rain, temperature, altitude, vegetation, and land use could identify new at-risk areas where CHIKV circulation could be confirmed by local serosurveys.

The continued geographical spread of CHIKV, through travel and trade, to temperate areas with favorable virus-vector-vertebrate and environmental conditions is conceivable. Since isolated cases and outbreaks have already been reported in the United States and Europe [2, 9, 10], it is important to focus future work on identifying potential factors that might help limit the spread of CHIKV, exploring therapeutic options for both acute and chronic disease, and developing a global strategy to combat the progression of the disease.

We have gained more understanding of the epidemiology and clinical spectrum of the disease, owing to the recent outbreaks in Africa and Asia. These lessons learned will help the Americas not only to better understand the acute and chronic phases of the disease, but also to better follow up at-risk population groups**.**
